# Impact of Surgical Timing, Fasciotomy, and External Fixation on Infection Risk in Tibial Plateau Fractures

**DOI:** 10.3390/jpm15030108

**Published:** 2025-03-11

**Authors:** Salvatore Risitano, Antonio Rea, Giorgia Garofalo, Francesco Onorato, Ahmed Elzeiny, Stefano Artiaco, Marcello Capella, Pier Francesco Indelli, Alessandro Massè

**Affiliations:** 1 Department of Orthopaedic Surgery and Traumatology, “Città Della Salute e Della Scienza”, CTO, 10126 Turin, Italy; stefano.artiaco@unito.it (S.A.);; 2 Institute of Biomechanics, Paracelsus Medical University (PMU), 5020 Salzburg, Austria; pindelli@stanford.edu; 3 Department of Orthopaedic Surgery and Traumatology, Ospedale Santa Croce e Carle, 12100 Cuneo, Italy; antoniorea.med@icloud.com; 4 Department of Orthopaedic Surgery and Traumatology, Ospedale degli Infermi, 10098 Rivoli, Italy; 5 Department of Orthopaedics, Traumatology and Rehabilitation, University of Turin, 10120 Turin, Italy; francesco.onorato@unito.it; 6 Department of Orthopaedics and Traumatology, Faculty of Medicine, Kafr El Sheikh University, Kafr El Sheikh 33516, Egypt; elzeiny1890@gmail.com; 7 Department of Orthopaedic Surgery, Südtiroler Sanitätsbetrieb, 39042 Brixen, Italy; 8 Department of Orthopaedic Surgery, Stanford University School of Medicine, Redwood City, CA 94061, USA

**Keywords:** tibial plateau fractures, infection, external fixator, risk factors, arthroscopy-assisted fixation

## Abstract

**Background/Objectives**: Tibial plateau fractures (TPFs) are commonly associated with complex patterns requiring advanced surgical strategies. High-energy trauma often results in severe soft tissue damage, complicating surgical outcomes. Despite advancements in soft tissue management, postoperative complications such as surgical site infections (SSIs) remain prevalent, with rates ranging from 9.9% to 30%. This study aims to analyze risk factors and surgical approaches influencing acute SSIs following TPF fixation. **Methods**: A retrospective analysis was conducted on 365 patients treated for TPFs with open or arthroscopy-assisted reduction and internal fixation (ORIF/ARIF) at a single center between January 2018 and December 2023. Inclusion criteria encompassed fractures classified by the Schatzker system and definitive management through ORIF/ARIF. Exclusion criteria included non-tibial plateau fractures, polytrauma, multiligament injuries and associated femoral fractures. Patient demographics, fracture patterns, surgical interventions, and postoperative complications were reviewed. Statistical analysis was performed using chi-square and ANOVA tests, with significance set at *p* < 0.05. **Results**: The final cohort included 364 patients (mean age: 45.4 ± 17.4 years; 59.2% male). High-energy fractures (Schatzker IV–VI) accounted for 47.7%, with 6.86% being open fractures. The mean interval to surgery was 14.9 ± 20.6 days. Superficial infections occurred in 21 cases (5.8%), predominantly at external fixator pin sites, while 15 cases (4.1%) involved deep infections. A statistically significant correlation was observed between SSIs and preoperative fasciotomy (*p* < 0.0001), damage control orthopedic protocols (*p* < 0.0001), and delays in definitive treatment of 10–30 days (*p* < 0.0001). No significant associations were found between infection rates and fracture type, dual surgical approaches, or the use of arthroscopy. **Conclusions**: External fixation, preoperative fasciotomy, and delayed definitive treatment are independent risk factors for SSIs following TPF fixation. High-energy injuries and soft tissue damage exacerbate infection risk. A personalized surgical approach, based on minimally invasive techniques and optimized surgical timing may mitigate these complications and significantly improve clinical outcomes in TPFs.

## 1. Introduction

Tibial plateau fractures (TPFs) account for 1.6% of all fractures in adults, and approximately 18.6% of tibial fractures [1]. These injuries take place when a varus or valgus force, combined with axial loading, is applied to the knee [2]. The incidence of TPFs follows a bimodal distribution, with high-energy trauma predominantly affecting younger individuals and low-energy trauma commonly observed in elderly or osteoporotic patients [3].

Due to the complexity of TPF fracture patterns, various classification systems have been proposed in the literature. Historically, the Schatzker classification system, introduced in 1974, has been the most widely used. This system categorizes TPFs into six subtypes based on fracture pattern, mechanism of injury, and severity as assessed by two-dimensional imaging [4]. However, traditional two-dimensional TPF classifications have been progressively being supplanted by three-dimensional approaches, such as the ten-segment classification or the three-column model [5,6]. The primary goals of TPF treatment are to restore the articular surface and re-establish the mechanical and anatomical axis of the limb.

The gold standard surgical option is osteosynthesis through locking plates which allow adequate internal stability and early knee mobilization. However, in case of open fractures or extensive soft tissue damage, a definitive treatment through an external fixator should be considered [7,8].

High-impact trauma resulting in Schatzker types IV to VI fractures is often associated with soft tissue injuries and poorer functional outcomes [9,10].

Complication rates following surgical treatment of TPFs have been reported to range from 26% to 33%, with variability influenced by patient and injury-related factors [9,11,12]. Among these complications, the most common is infection, with an incidence reported as high as 23.2–30% [13]. However, the advances in surgical approaches and techniques have significantly improved complication rates [14]. For example, damage control orthopedic (DCO) strategies using a temporary external fixator have improved soft tissue management prior to definitive surgery. Furthermore, the adoption of minimally invasive techniques and innovations in implant materials have improved periosteal preservation and healing outcomes [15,16].

Arthroscopy has recently emerged as a valuable tool in managing TPFs, with no significant differences in complication rates observed when comparing traditional open reduction internal fixation (ORIF) with arthroscopy-assisted internal fixation (ARIF) [17]. Although complications following surgical management of TPFs cannot be entirely eliminated, a deeper understanding of the risk factors influencing the outcomes could optimize management strategies and further reduce complication rates in the acute setting. The aim of the present study was to analyze complications and risk factors associated with TPFs to assess whether injury characteristics or surgical approaches could predict acute postoperative surgical site infections (SSIs) or unplanned outcomes.

## 2. Materials and Methods

Institutional review board approval was obtained prior to conducting chart review and analysis. This retrospective, single-center review was conducted using the institutional health platform (TrakCare, Intersystem, Cambridge, MA, USA). Data were collected on all TPFs presented to the emergency department of a single academic institution between 1 January 2018, and 31 December 2023. Cases were identified by cross-referencing diagnosis and treatment codes according to the ICD-10 standard classification system. The inclusion criteria were based on the following ICD-10 codes: proximal tibial epiphyseal fractures, both open and closed (823.00, 823.02, 823.10, 823.12), and ORIF procedures (79.36, 79.16).

The initial query identified 548 patients. This dataset was further refined by cross-referencing with codes for fasciotomy procedures and external fixator applications (83.14, 78.17). Each patient’s clinical history, emergency department presentation, physical and radiological findings, injury management, hospitalization, and follow-up in outpatient clinics were thoroughly reviewed. Each fracture was classified according to the original Schatzker classification system.

Exclusion criteria included patients with fractures at locations other than the tibial plateau, fasciotomy performed in regions other than the leg, polytrauma patients who underwent damage control orthopedics (DCO) outside the leg, wrongly coded fractures and adolescent patients with open growth plates. Following the application of these criteria, 412 patients were included in the final analysis. Additional exclusions included patients treated non-operatively, those who received definitive management other than ORIF or ARIF, patients with associated multiligament knee injuries (Schenck V), isolated bony avulsions of the tibial plateau, fractures not classifiable by the Schatzker system, and associated distal femoral fractures.

We ultimately identified 365 patients who underwent ORIF or ARIF for TPFs. Charts were reviewed for demographic data, imaging modalities, fracture patterns, surgical procedures preceding definitive surgery, time to definitive surgery, intraoperative data including implants utilized, and clinical course including return to the operating room for all etiologies. Postoperative complications at surgical sites occurring within the first year after surgery were also reviewed. One patient was excluded because of missing postoperative controls. All operations were performed under spinal or general anesthesia, in an operating room with laminar flow, by 5 senior surgeons. In case of bi-condylar fractures a double approach medial and lateral was performed with at least two tibial columns fixation. All patients received standard prophylactic antibiotics. All patients received therapeutic dosing for antithrombotic prophylaxis for at least 30 days postoperatively. Patients were made weight bearing as tolerated from 60 to 90 days postoperatively and all patients participated in physical therapy with non-weight bearing passive and active range of motion (ROM) exercises from postoperative day 1. All sensitive data were managed in a pseudo-anonymized format to ensure patient confidentiality.

## 3. Results

A total of 364 patients were included in the study population. The mean age of the patients was 45.41 ± 17.4 years (range 14–86), with 59.2% being male, and without major differences regarding the affected side. CT scan was used to assess fracture patterns in 315 (86.5%) patients, while associated MRI was used to identify any ligamentous and meniscal injuries in 56 (15.4%) cases.

The mean interval between the trauma and the surgery was 14.9 ± 20.6 days, ranging from the same day to a maximum delay of 219 days. Within the cohort included, 109 (29.9%) patients underwent a two-stage approach, with the initial application of DCO with external fixator followed by subsequent definitive synthesis. Twenty (5.5%) patients were complicated due to compartment syndrome and therefore underwent urgent fasciotomy of which 16 (4.4%) were performed within 12 h of the damage control, while four (1.1%) were performed after more than 12 h. Concerning timing with respect to definitive treatment, 15 (4.1%) fasciotomies were performed before and five (1.4%) after the definitive surgery. The mean interval between the emergency arrival and the fasciotomy was 26.5 ± 144.7 min, while the interval between the emergency arrival and damage control was 13.1 ± 65.9 min.

Regarding fracture characteristics, 47.7% were high-energy fractures (Schatzker types IV, V, and VI) and 25 were open fractures (6.86%). Most of the fractures were Schatzker type II and type VI (55.4% of the patients), while according to AO classification half of the fractures were type B3 and C3 (51% of the patients). A detailed preoperative patient’s demographic is shown in Table 1 and classification according to Schatzker classification is reported in Table 2.

TPF definitive treatment was performed through percutaneous fixation with cannulated screws in 165 (45.2%) patients; however, the rest of the patients underwent fixation through single or double approaches using plates and screws. Arthroscopy assistance was used in 135 (37%) of patients for accurate articular surface reduction. A detailed summary of surgical approaches is reported in Table 3.

Regarding the rate of infection related to TPFs, 36 cases (9.9%) suffered from SSIs after TPFs fixation. In particular, 21 (58%) patients presented a superficial site infection, of which 13 were located at the external fixator pin entry points. All patients were treated exclusively with empiric intravenous antibiotics with successful healing, with the exception of one patient who underwent wound revision.

The remaining 15 patients (32%) presented deep surgical infection; all started empiric intravenous antibiotic therapy immediately after the completion of intraoperative microbiological sampling. Target antibiotic therapy was then started after culture-positive samples. Ten patients had plates and screws removed and were converted to circular external fixators, while five of them continued antibiotic therapy up to bone healing.

Among the 109 (29.9%) patients who underwent damage control protocol, 21 patients showed postoperative infection, demonstrating a statistically significant correlation between incidence of infection and DCO (*p* < 0.0001) as detailed in Table 4.

**Figure 1:**
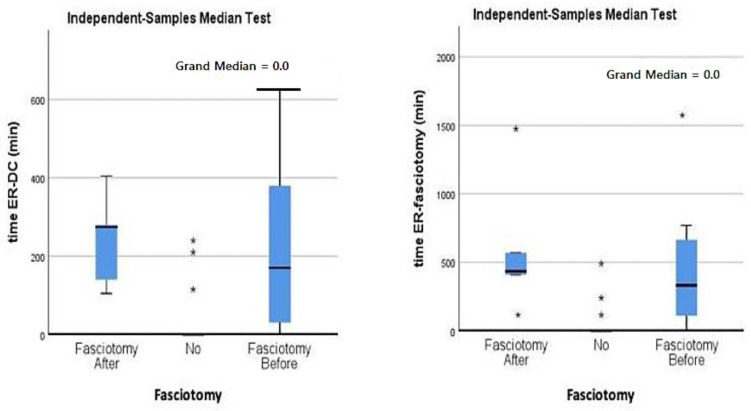
Comparison of medians across study groups demonstrating a statistically significant correlation between post-operative infection and DCO. Time between emergency department presentation and the performed DCO or fasciotomy did not correlate with infection rate (*p* > 0.05). Star symbol (*): distribution of isolated cased; no fasciotomy and two outliers (one in the “fasciotomy after” and another in the “fasciotomy before” category.

Within the 15 patients who underwent preoperative fasciotomy, five patients (33.3%) showed postoperative infection, demonstrating a statistically significant correlation both when performed with DCO or after DCO (*p* < 0.0001) as detailed in Figure 1. However, the median elapsed time between emergency department presentation and the performed DCO or fasciotomy did not correlate with infection rate (*p* > 0.05).

**Figure 2:**
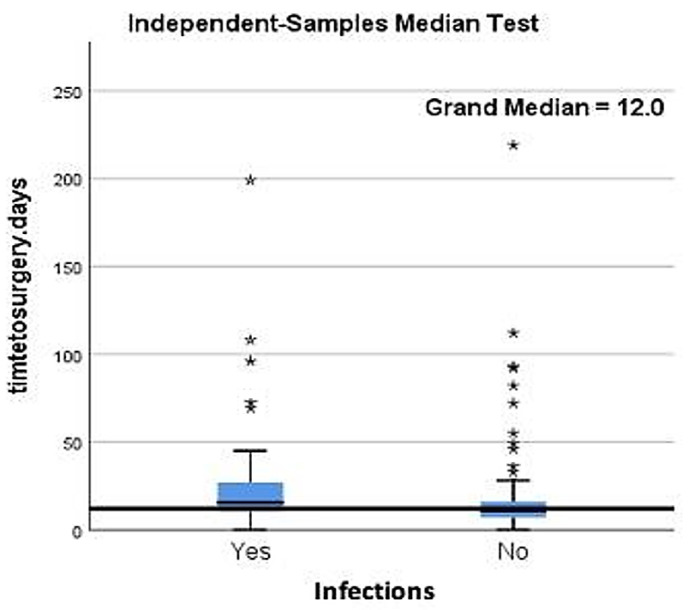
Comparison of medians across study groups demonstrating a significant correlation between delay in definitive treatment and incidence of infection (*p* < 0.0001). Star symbol (*): distribution of ouliers for infections and non infections.

A significant correlation was encountered between delay in definitive treatment and incidence of infection, especially when time till surgery was between 10 days and 30 days from the initial trauma (*p* < 0.0001) as shown in Figure 2. Fracture pattern, degree of exposure, number of surgical access incisions, and arthroscopic assistance did not statistically correlate with infection rate.

## 4. Discussion

The primary finding of this clinical study was that preoperative fasciotomy, DCO with external fixator, and delays in definitive surgical treatment are positively correlated with SSIs. TPFs are among the most common lower extremity injuries and are frequently associated with severe soft tissue disruptions, placing them at a relatively high risk of postoperative SSIs. Historically, infection rates as high as 80% were reported in older studies [18]. Although improved soft tissue management and less invasive techniques have significantly reduced SSI rates, these injuries remain prone to complications [19]. Previous research has identified various nonsurgical risk factors for SSIs, including age, diabetes, smoking, body mass index, intensive care unit stays, Morel–Lavallée lesions, number of operations, congestive heart failure, contaminated surgeries, chemoprophylaxis, prolonged preoperative hospital stays, and steroid use [20]. In the current study, we aimed to evaluate the impact of surgical treatment variables on TPF outcomes.

However, it is important to note that the wide range of patient characteristics and fracture management strategies limits the ability to establish definitive evidence regarding optimal treatment protocols. Furthermore, the lack of a standardized definition of postoperative infection adds complexity. To address this problem, the European Bone and Joint Infection Society defined criteria for infection related to fractures, providing a standardized definition for post-osteosynthesis infections [21]. Despite these challenges, our observed infection rate after TPF osteosynthesis of around 10% was comparable to the previous recent literature. Shao et al. reported a similar SSI incidence in a meta-analysis of 2214 TPF cases treated with ORIF, identifying risk factors such as open fractures, compartment syndrome, operative time, tobacco use, and external fixation [22]. A recent meta-analysis reported approximately 6% risk of deep infection for these kinds of injury when treated through ORIF, increasing to 15% when including deep, superficial, and pin-site infections [23].

In our cohort, 21 out of 109 patients (29.9%) treated with a DCO protocol developed postoperative infections. Statistical analysis revealed a significant correlation between SSIs and initial osteotaxis using external fixation. This finding warrants careful interpretation. In the context of high-energy trauma, external fixators allow for careful monitoring of skin conditions and appropriate evaluation of the timing for definitive surgery. Furthermore, optimal pin placement, considering the subsequent surgical approach, along with proper pin care, is essential to reduce the risk of infection. For instance, Egol et al. reported a low incidence of deep infection through a temporary spanning ExFix for tibial plateau fractures [24]. Conversely, other studies found no association between external fixation and deep infections [25,26].

It has been well established that high-energy TPFs with greater soft tissue injuries are associated with a high rate of complications due to the multiple risk factors such as extensive soft tissue damage, complex fractures, open injuries, and compartment syndrome [27]. In addition, these patients often require longer surgeries, prolonged hospital stays, and multiple debridements to ensure proper soft tissue healing, which inherently increases infection risk. Consequently, it is challenging to determine whether spanning external fixation independently elevates SSI risk or is simply associated with more severe injuries. Our study confirmed that high-energy fractures requiring external fixation are linked to higher SSI rates. Parkkinen et al. reported that the use of an external fixation increases the odds of postoperative infection by 3.8 to 5.4 times, rising to 11.2 in bicondylar fractures [28].

Concerning the removal of the temporary fixators before the surgery, the literature reported no statistical difference in deep infection rates whether the fixator is fully removed or retained, or whether the entire device or only the pins are sterilized [29,30].

In this study, the mean interval between the trauma and the surgery was 14.9 ± 20.6 days. We observed that longer delays between trauma and surgery, particularly intervals of 10–30 days, were significantly associated with higher postoperative infection rates (*p* < 0.0001). High-energy injuries often present soft tissue conditions unsuitable for immediate surgery [31]. We intentionally decided to exclude polytrauma patients who underwent DCO or fasciotomies outside the leg to exclude all those patients where the surgical treatment could have been the original source of the infection. However, polytraumatic patients and high-urgency comorbidities could be another cause of delay in definitive treatment.

Compartment syndrome is a well-recognized complication of TPFs, with reported incidences ranging from 7.3% to 27% in bicondylar fractures [14,25]. In the current study, 5.5% of patients required fasciotomies for compartment syndrome, which was identified as an independent risk factor for postoperative SSIs (*p* = 0.003). This aligns with findings by Momaya et al. who reported a deep infection rate of 8.3% in patients with compartment syndrome [26] and Morris et al. who similarly observed an increased risk [25]. In contrast, Lin et al. found no significant association between compartment syndrome and SSIs [32].

Arthroscopy-assisted TPF reduction was performed in 135 patients (37%), primarily in Schatzker II, III, and IV fractures, without an associated increase in infection rates. The use of arthroscopy associated with TPF osteosynthesis is an increasingly growing practice. It allows direct observation of the reduction at an intra-articular level, as well as the diagnosis of possible associated joint injuries. Although its use in high-grade TPFs remains debated due to potential complications such as acute compartment syndrome, recent studies suggest that arthroscopy does not increase complications in either low-grade or high-grade fractures [33,34]. In our study, no cases of compartment syndrome were reported with the use of arthroscopy.

The number of surgical incisions and plates has also been debated as a potential risk factor for infection [14,35]. Dual incisions may involve extensive soft tissue dissection, increasing infection risk. In our study, dual-incision approaches were used in 159 patients (43.6%), with no significant association between this approach and SSIs. Similarly, Momaya et al. reported that the double incision approach in 141 (26.6%) cases, with an associated two plates in most of the cases, was not associated with increased infection [26]. Conversely, Morris et al. showed that fractures requiring the use of two incisions and two plates were at increased risk for infection [25].

This study has several limitations. As a retrospective analysis, it is subject to recall bias. However, clinical data were meticulously recorded in an institutional health platform, mitigating this limitation. The study lacks long-term functional outcomes which would provide completeness to the results. Furthermore, different variables and comorbidities such as trauma severity scores that may have affected wound healing have not been collected. However, the primary objective of this study was to evaluate the SSIs according to injury characteristics or surgical approaches rather than patient demographics. Finally, all the temporary spanning with external fixators, fasciotomies, and the definitive surgical procedures for these fractures were performed by different surgeons, with different surgical management criteria which could have conditioned the outcomes.

## 5. Conclusions

TPFs pose a complex therapeutic challenge, with postoperative complications influenced by factors such as delays in definitive treatment, use of external fixators, and the need for fasciotomies. Personalized surgical decision-making, tailored to soft tissue damage, injury characteristics, and surgical approaches, can significantly improve clinical outcomes.

## Figures and Tables

**Table 1:** Preoperative patient’s demographic.

Age at surgery (years)	45.4 ± 17.4 (range: 14 to 86)
Gender (female/male)	148 (40.7%)/216 (59.3%)
Side (left/right)	206 (56.6%)/158 (43.4%)
Associated fractures (yes/no)	186 (51.1%)/178 (48.9%)
Open fracture (yes/no)	25 (6.86%)/339 (93.14%)
CT (yes/no)	315 (85.5%)/49 (13.5%)
MRI (yes/no)	56 (15.4%)/308 (84.7%)
Time to surgery (days)	14.86 ± 20.5 (0–219)
Time ER–fasciotomy (min)	26.44 ± 144.7 (0–1570)
Time ER–DC (min)	13.07 ± 65.8 (0–665)

CT: computer tomography, MRI: magnetic resonance imaging, ER: emergency room, DC: damage control.

**Table 2:** Fractures’ Schatzker classification.

Schatzker	Frequency	Percentage
I	35	9.6
II	121	33.2
III	35	9.6
IV	57	15.6
V	36	9.9
VI	81	22.2

**Table 3:** Surgical approaches.

DCO (yes/no)	109 (29.9%)/255 (70.1%)
Fasciotomy (yes/no) With DCO/After DCO Before DS/After DS	19 (5.2%)/345 (94.8%) 15 (4.1%)/4 (1.1%) 14 (3.8%)/5 (1.4%)
DS percutaneous screws	165 (45.3%)
DS single approach	41 (11.3%)
DS double approach	79 (21.7%)
DS triple approach	68 (18.7%)
DS quadruple approach	11 (3%)

DCO: damage control orthopedics, DS: definitive surgery.

**Table 4:** DCO and infection.

	Patients	Infection	Degree of Freedom	Mean Square	F Value	Significance
With DCO	109	21	1.402	1.402	16.349	*p* < 0.0001
Without DCO	255	15	31.038	0.086		

DCO: damage control orthopedics.

## Data Availability

The raw data supporting the conclusions of this article will be made available by the authors on request.

## Abbreviations

The following abbreviations are used in this manuscript:

TPFs	Tibial Plateau Fractures
SSIs	Surgical Site Infections
DCO	Damage Control Orthopedics
ORIF	Open Reduction Internal Fixation
ARIF	Arthroscopic Assisted Internal Fixation
CT	Computed Tomography
MRI	Magnetic Resonance Imaging
